# Oncogenic Gene CNOT7 Promotes Progression and Induces Poor Prognosis of Glioma

**DOI:** 10.1007/s12033-024-01223-5

**Published:** 2024-07-10

**Authors:** Feng Lu, Xiulong Jiang, Kun Lin, Pengfeng Zheng, Shizhong Wu, Guangming Zeng, De Wei

**Affiliations:** 1 Shengli Clinical Medical College of Fujian Medical University, 134 East Street, Fuzhou, 350001 China; 2 Department of Neurosurgery, Fuzhou University Affiliated Provincial Hospital, 134 East Street, Fuzhou, 350001 China; 3 Department of Neurosurgery, Shengli Clinical Medical College of Fujian Medical University, 134 East Street, Fuzhou, 350001 China; 4 Department of Neurosurgery, Fujian Provincial Hospital, 134 East Street, Fuzhou, 350001 China

**Keywords:** CNOT7, Glioma, HDAC2

## Abstract

Glioma is the most common malignant brain tumor in the central nervous system with the poor prognosis of patients. The CNOT7 (CCR4-NOT Transcription Complex Subunit 7) is an important functional subunit of CCR4-NOT protein complex that has not been reported in glioma. In this study, we aimed to explore the function of CNOT7 in glioma. The TCGA (The Cancer Genome Atlas) and CGGA (Chinese Glioma Genome Atlas) databases were used for investigating the expression and survival condition of CNOT7 in glioma. The cellular function experiments of qRT-PCR, CCK-8 assays, wound healing assays, and Transwell assays were conducted to verify the function of knockdown CNOT7 in the glioma cell lines DBTRG and U251. Enrichment analysis was used to explore the molecular mechanism of CONT7 in glioma. What is more, the upstream regulation transcription factors of CNOT7 were analyzed based on the ChIP-Atlas and cBioportal (provisional) databases, and verified by the qRT-PCR and luciferase reporter assay. The CNOT7 was highly expressed in glioma and presented the poorer prognosis. The knockdown of CNOT7 inhibited the proliferation, migration, and invasion of glioma cell line, compared to control group. The enrichment analysis revealed that the CNOT7 participated in the development of glioma via G2M checkpoint, E2F targets, IL6-JAK-STAT3, and TNF-α signaling pathways via NF-κB. Besides, it was found that the HDAC2 (Human histone deacetylase-2) contributes to increased CNOT7 expression in glioma. The high-expressed CNOT7 is an oncogene with poor prognosis and participate the progression of glioma.

## Introduction

Glioma is the most common tumor of the central nervous system (CNS), accounting for about half of the adult intracranial tumors, and it mainly comes from glial cells [1, 2]. According to the differentiation degree of glial cells and histopathological characteristics, the WHO (World Health Organization) Central Nervous System Tumor Grading Guidelines in 2007 classified glioma into 1–4 grades. The Grade I glioma and Grade II are classified as Lower Grade Glioma (LGG) [3, 4]. Grade IV glioblastoma, also known as polymorphous glioblastoma (GBM), has the highest incidence rate, accounting for 46.1% of all gliomas. Its annual incidence rate is about 6.6/100000, and its 5-year survival rate is less than 10% [5, 6]. The pathogenesis of glioma is complex, involving many aspects of the patient’s genetic background, gene mutation, tumor microenvironment, and so on [7]. Due to the limitations of surgery and radiotherapy, more and more studies are devoted to the breakthrough of GBM-related pathogenic genes. Targeted gene therapy of GBM has gradually become the trend of future research.

In recent years, the research on gene markers of glioblastoma mainly focuses on the exploration of diagnostic, therapeutic, and prognostic markers [8]. The markers found related to the diagnosis and prognosis of glioblastoma include O6-methylguanine-DNA methyltransferase (MGMT) [9], epidermal growth factor receptor (EGFR) [10], isocitrate dehydrogenase 1/2 (IDH1/2) [11], 1p/19q deletion [12], etc. The CNOT7 is an important functional subunit of CCR4-NOT protein complex [13]. The function of CNOT7 that degrading mRNA and regulating gene transcription through deadenylation has been widely confirmed [14]. CNOT7 has been reported to be related to some tumors, such as ovarian cancer [15], breast cancer [16], and colorectal cancer [17]. The underlying mechanism of CONT7 in cancer has been reported in some studies. For instance, CNOT7 modulates biological functions of ovarian cancer cells via AKT signaling pathway [15]. CNOT7 ubiquitinate and accelerate decay of SOCS3 mRNA to suppressing hepatocellular carcinoma cell migration in vitro and metastasis in vivo [18]. High expression of CNOT7 drives tumor cell autonomous metastatic potential, which requires its deadenylase activity [19]. However, the expression and function of CNOT7 in glioma has not been reported yet.

In this study, we aimed to explore the function of CNOT7 in glioma. The TCGA and the CGGA database were used for investigate the expression and survival condition of CNOT7 in glioma. The cellular function experiments of qRT-PCR, CCK-8 assays, wound healing assays, and Transwell assays were conducted to verify the function of knockdown CNOT7 in the glioma cell lines DBTRG and U251. The molecular mechanism of CONT7 in glioma was analyzed by the GSEA and GSVA enrichment analysis. What is more, the upstream regulation transcription factors of CNOT7 were analyzed based on the ChIP-Atlas and cBioportal (provisional) database, and verified by the qRT-PCR and luciferase reporter assay. This research provides reference for clinical diagnosis and treatment to explore new therapeutic targets for glioma.

## Materials and Method

### Data Acquirement and Procession

The expression level and overall survival of CNOT7 in GBM and LGG were obtained from TCGA database and analyzed by GEPIA online website (http://gepia.cancer-pku.cn/). The expression level and all WHO grade survival of CNOT7 in different WHO grade, IDH mutation status, and 1p/19q co-deletion status were obtained and visualized by the CGGA database (http://www.cgga.org.cn/). The histone and transcription factors that binding to CNOT7 were analyzed based on the ChIP-Atlas database. The co-expression of CNOT7 with TOP2A or HDAC2 in glioma patients’ samples was obtained from cBioportal (provisional) database (http://www.cbioportal.org/).

### Cell Line and Transfection

Human glioblastoma cell lines U251 and DBTRG were purchased from Shanghai Institute of Cell Biology, Chinese Academy of Sciences. U251 and DBTRG cells were routinely cultured in DMEM medium containing 10% fetal bovine serum at 5% CO_2_ and 37 °C. Cells in logarithmic growth phase were used in various experiments. The CNOT7 shRNAs, HDAC2 shRNA, and negative control (NC) were chemically synthesized by shanghai Sangon (Shanghai, China). CNOT7 shRNA, negative control, and HDAC2 shRNA were transfected according to the product specification into the cells using Lipofectamine 2000 reagent (Invitrogen). The experiment was performed 3 times (biological replicates). The sequence of shRNAs that used in this research is listed below:

- CNOT7-shRNA1: 5′-TTGTGTCGCTGAGCTCGCGCT-3′
- CNOT7-shRNA2: 5′-AAATATTTTATCCTTTATTTATGT-3′
- CNOT7-shRNA3: 5′-GTCTTGCAACCACACCTGGAAA-3′
- HDAC2-shRNA: 5′-AAGCATCAGGATTCTGTTA-3′

### QRT-PCR Detection

The total RNA of the sample was extracted with TRIZOL. Reverse transcript the RNA sample obtained in the previous step to obtain the corresponding cDNA. Dilute the synthesized primer to a concentration of 10 μM. Perform qRT-PCR detection (reaction conditions: 95 °C 10 min, 60 °C 20 s × 40 cycles, 72 °C 30 s, 4 °C 5 min). GAPDH served as an internal control. The experiment was performed 3 time (biological replicates). The primer sequence is as follows:

- CNOT7-Forward: 5′-ATGCCAGCGGCAACTGTAG-3′
- CNOT7-Reverse: 5′-TCGGTGTCCATAGCAACGTAA-3′
- HDAC2-Forward: 5′-ATGGCGTACAGTCAAGGAGG-3′
- HDAC2-Reverse: 5′-TGCGGATTCTATGAGGCTTCA-3′
- GAPDH-Forward: 5′-GGAGCGAGATCCCTCCAAAAT-3′
- GAPDH-Reverse: 5′-GGCTGTTGTCATACTTCTCATGG-3′.

### CCK-8 Assays

U251/DGBRT and its stably transfected cell lines were cultured in cell incubator. The cell count plate counts the number of cells in the prepared cell suspension, and inoculates the cells in the 96-well culture plate. The number of cells added in each well is 3 × 10^3^. In the experiment, each group was set with 5 multiple wells, and then added with CCK-8 solution after incubation for 0 h, 24 h, 48 h, and 72 h respectively. Then, the culture plate was placed at 37 °C, and the value of OD450 was detected after incubation for 1 h in a 5% CO_2_ incubator. The experiment was performed 3 times (biological replicates).

### Wounding Healing Assays

The U251 and DGBRT cell were seeded in 6-well plate after transfection. At 48 h, a line was drawn in the hole with the 200 mL pipette. The scribed cells were cleaned with PBS, and fresh medium was added into cells. The cells were put into the incubator. The migration cell images were taken and recorded under the microscope at 0 h and 48 h. The experiment was performed 3 times (biological replicates).

### Transwell Assays

U251/DGBRT and its stably transfected cell lines were cultured in cell incubator.

Matrigel matrix adhesive was diluted with 1:8 and coated with the upper surface of the transwell cell bottom membrane, and dried for 3 h. Aspirate the residual liquid in the culture plate and add 50 to each hole μL 10 g/L BSA serum-free culture medium, incubation at 37 °C for 30 min. Taking 200 μL cell suspension is added to the upper chamber of Transwell cell and 600 cells are added to the lower chamber of 24-well culture plate μL containing 10% FBS medium. The culture plate was placed in a CO_2_ incubator at 37 °C for 48 h. Take out the chamber, fix it in a 24-hole plate with 4% paraformaldehyde (or 95% alcohol) for 20 min, and dye it with crystal violet solution for 15 min. ut the sample under the inverted microscope and take photos. The experiment was performed 3 times (biological replicates).

### GSEA and GSVA Analysis

Gene Set Enrichment Analysis (GSEA) analysis was performed on the high and low expression groups of CNOT7 based on TCGA GBM dataset through GSEA3.0 software to obtain the HALLMARK signal pathway related to glioma. All genes were divided into CNOT7 high expression and low expression groups. HALLMARK pathway gene set was downloaded from Msigdb. GSEA analysis was performed by GSEA functions in the clusterProfiler package. Gene Set Variation Analysis (GSVA) analysis was also performed based on the TCGA GBM dataset, either. The analysis was carried out via R package GSVA (version 3.6.1).

### Luciferase Reporter Gene Experiment

U251 cells were seeded into 96-well plate. The amplified CNOT7 3′-UTR-WT and corresponding CNOT7 3′-UTR-MUT were cloned into pGL3 luciferase vector (Promega Corporation). U251 cells were co-transfected with CNOT7 3′-UTR-WT or CNOT7 3′-UTR-MUT and HDAC2 shRNA using Lipofectamine 3000. Luciferase assays were conducted 48 h after transfection using the Dual-luciferase Reporter assay system (Promega). The experiment was performed 3 times in triplicate.

### Statistics Analysis

The experimental data obtained in this study have been repeated at least 3 times, and are expressed in the form of mean ± standard deviation. The data were statistically analyzed with SPSS 17.0. Morphological results were analyzed by software related to instruments and equipment. Data of two treatment groups in the same experiment were analyzed by paired *t* test. *P* < 0.05 means statistically significant.

## Results

### CNOT7 is Highly Expressed with Poor Prognosis in Glioma

**Fig. 1:**
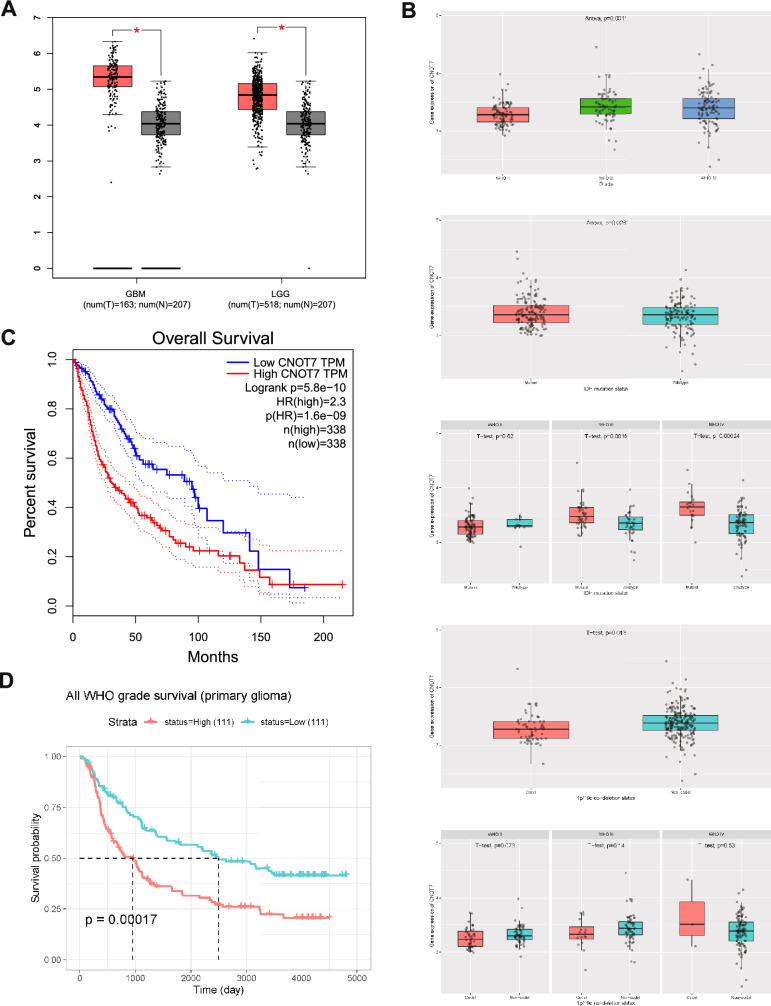
CNOT7 is highly expressed with poor prognosis in glioma. **A** The expression level of CNOT7 in Glioblastoma multiforme (GBM) and Brain Lower Grade Glioma (LGG) were higher than normal tissue based on The Cancer Genome Atlas (TCGA) database. **B** The expression level of CNOT7 in different WHO grade, IDH mutation status, and 1p/19q co-deletion status were obtained from the Chinese Glioma Genome Atlas (CGGA) database. **C** The overall survival of CNOT7 in glioma based on TCGA database. **D** The all WHO grade survival of CNOT7 in primary glioma based on CGGA database

First, through the TCGA database, we found that CNOT7 presented higher expression level in Glioblastoma multiforme (GBM) or Brain Lower Grade Glioma (LGG) samples compared with normal samples (Fig. 1A). The CNOT7 was gradually increased in different WHO grades of glioma based on the CGGA database. Besides, the CNOT7 was higher expressed in IDH mutation samples compared to wildtype samples, especially in WHO grades III and IV. The CNOT7 was higher expressed in non-codel 1p/19q co-deletion status, especially in WHO grades II and III (Fig. 1B). As for the survival analysis of CNOT7, the higher expression of CNOT7 was present poorer prognosis in glioma (Fig. 1C and D). Together, these results indicated that CNOT7 is highly expressed with poor prognosis in glioma, which suggest that it might be a potential oncogene in glioma.

### The Knockdown of CNOT7 Suppresses the Proliferation, Migration, and Invasion of Glioma Cell

**Fig. 2:**
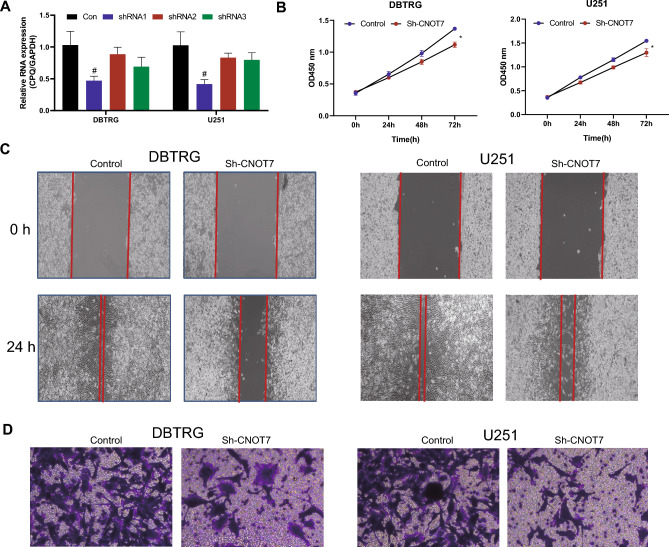
The knockdown of CNOT7 suppresses the proliferation, migration, and invasion of glioma cell. **A** The knockdown efficiency of CNOT7 shRNA 1–3 in DBTRG and U251 cell lines was detected by qRT-PCR. The shRNA 1 of CNOT7 was selected for the following experiments. **B** The effect of CNOT7 knockdown on proliferation of DBTRG and U251 cell lines were detected by CCK-8 assays. **C** The effect of CNOT7 knockdown on DBTRG and U251 cell lines were detected by wound healing assay. **D** The effect of CNOT7 knockdown on invasion of DBTRG and U251 cell lines were detected by Transwell assay. *N* = 3, *P < 0.05

In the next step, the function of CNOT7 was verified by cellular experiments. A total of 3 kinds of shRNA were designed and carried out for knockdown efficiency measurement. As illustrated in Fig. 2A, the shRNA-1 was presented the best knockdown efficiency in both DBTRG and U251 cell lines, and therefore, it was selected for the following experiments. Furthermore, the knockdown of CNOT7 inhibited the proliferation of DBTRG and U251 cell (Fig. 2B). The wound healing assays indicated that the knockdown of CNOT7 suppressed the migration of DBTRG and U251 cell (Fig. 2C). The invasion of DBTRG and U251 cell was also attenuated, while the CONT7 was knocking down (Fig. 2D). These results indicated that the knockdown of CNOT7 suppresses the proliferation, migration, and invasion of glioma cell.

### The Molecular Mechanism of CNOT7 in Glioma

**Fig. 3:**
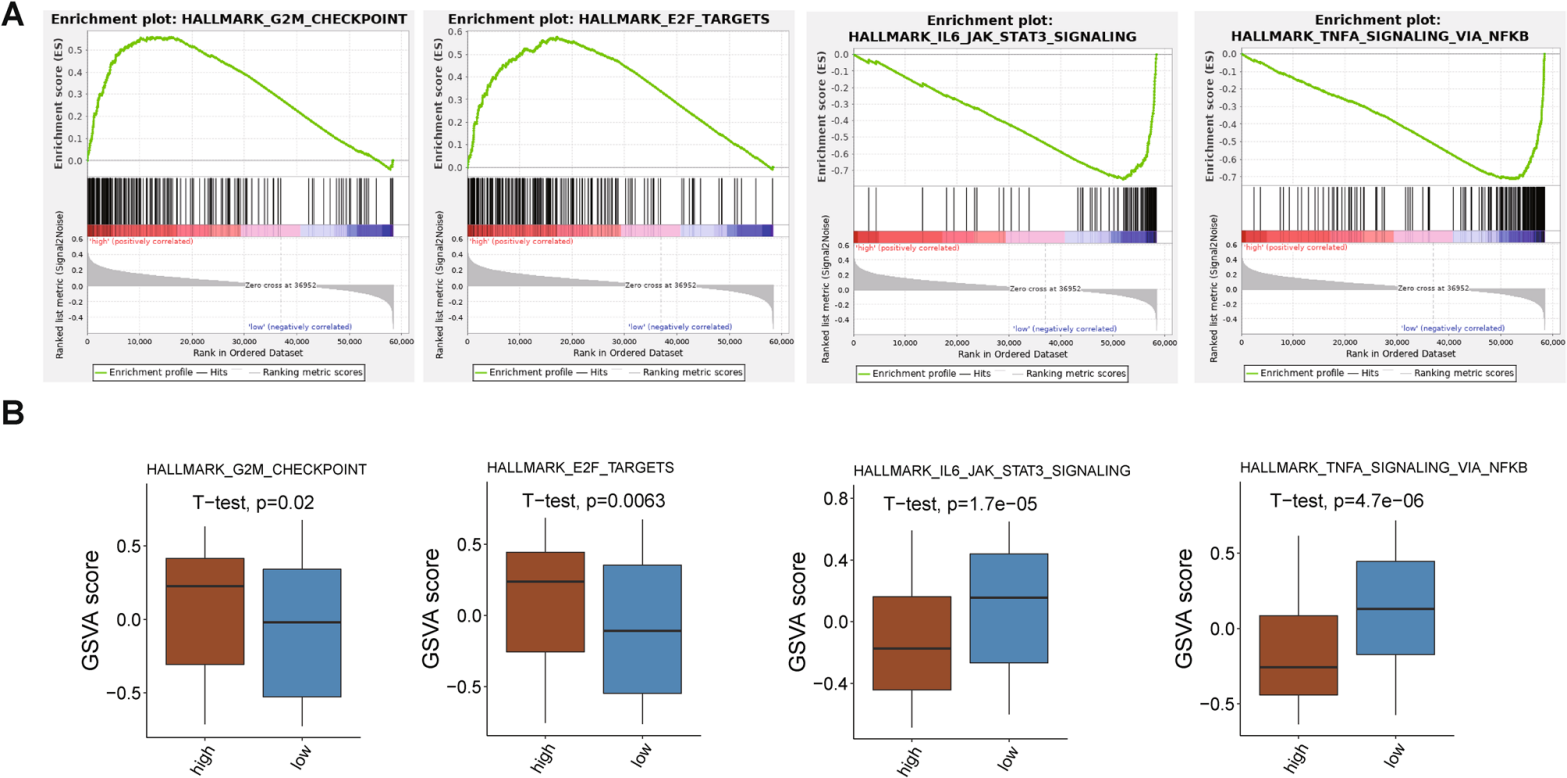
The molecular mechanism of CONT7 in glioma. **A** and **B** The GSEA (**A**) and GSVA (**B**) enrichment analysis revealed the molecular mechanism of CNOT7 with high or low expression in glioma based on HALLMARK signaling pathways gene set

Then, we sequentially explored the molecular mechanism of CNOT7 in glioma. The GSEA and GSVA enrichment analysis based on HALLMARK gene set was performed. As shown in Fig. 3A**,** the GSEA enrichment analysis revealed that the G2M checkpoint and E2F targets signaling pathways were active, while the CNOT7 was highly expressed. The IL6-JAK-STAT3 and TNF-α signaling pathways via NF-κB were inhibited, while the CNOT7 was lowly expressed. The results of GSVA were also consistent with that of GSEA (Fig. 3B). The G2M checkpoint [20] and E2F targets signaling pathways [21] were reported to promote the development of glioma. The IL6-JAK-STAT3 [22] and TNF-α signaling pathways via NF-κB [23] were associated with the inflammation in glioma. These results revealed that the CNOT7 was participated in the development of glioma via tumor-related signaling pathways.

### The HDAC2 Contributes to Increased CNOT7 Expression in Glioma

**Fig. 4:**
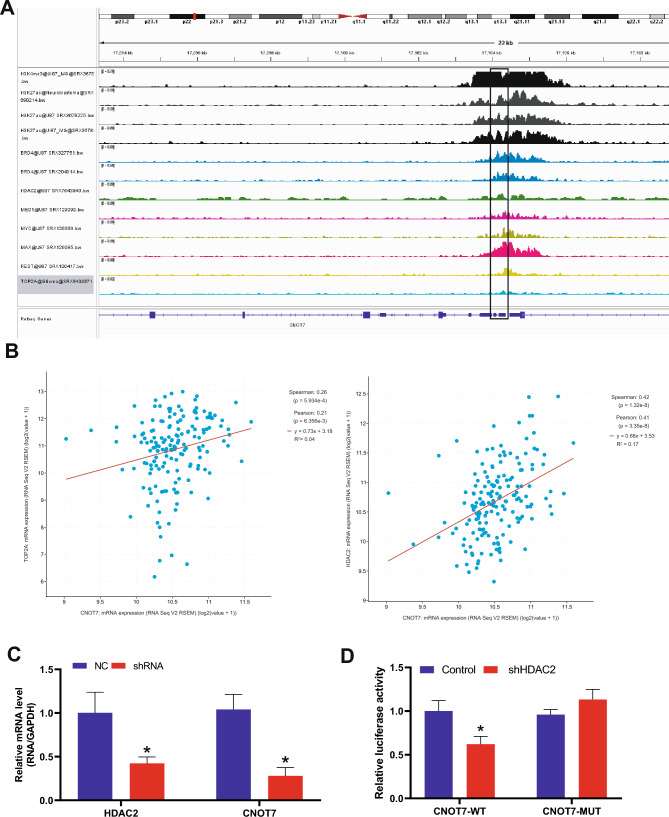
The HDAC2 contributes to increased CNOT7 expression. **A** The histone and transcription factors that binding to CNOT7 were analyzed based on the ChIP-Atlas database. **B** The CNOT7 was co-expressed with TOP2A or HDAC2 in glioma patients’ samples obtained from cBioportal (provisional) database. **C** The expression level of HDAC2 and CNOT7 in U251 cell line treated with HDAC2- shRNA was measured by qRT-PCR. **D** The binding of CNOT7 and HDAC2 was confirmed by luciferase reporter gene assay

Finally, we investigated the upstream regulation mechanism of CNOT7. The transcription factors of CNOT7 were analyzed based on the ChIP-Atlas database. As presented in Fig. 4A, some histones and transcription factors that might binding to CNOT7 were shown, such as H3K4me3, H3K27ac, BRD4, HDAC2, and TOP2A. To further verify the correlation of these histones or transcription factors with CNOT7, we analyzed the correlation of them with CNOT7 in cBioportal (provisional) database. As shown in Fig. 4B, the CNOT7 was positively correlated with transcription factors TOP2A or HDAC2 in glioma patients’ samples, especially HDAC2 (Spearmen = 0.42, Pearson = 0.41, *R*^2^ = 0.17). Therefore, HDAC2 was selected for further validation in vitro. We transfected the shRNA of HDAC2 into U251 cell, and the expression of HDAC2 and CNOT7 was both down-regulated, suggesting that the CNOT7 was regulated by HDAC2 (Fig. 4C). The binding of CNOT7 and HDAC2 was confirmed by luciferase reporter assay (Fig. 4D). These results indicated that HDAC2 contributes to increased CNOT7 expression in glioma.

## Discussion

In this research, it was the first time to investigate and verify the function of CCR4-NOT protein complex CNOT7 in glioma. According to the TCGA and CGGA databases, the CNOT7 was highly expressed in glioma and presented the poorer prognosis. The cellular function experiments indicated that the knockdown of CNOT7 inhibited the proliferation, migration, and invasion of glioma cell line. The analysis of GSEA and GSVA revealed that the CNOT7 participated in the development of glioma via G2M checkpoint, E2F targets, IL6-JAK-STAT3, and TNF-α signaling pathways via NF-κB. Finally, it was found that HDAC2 contributes to increased CNOT7 expression in glioma. These evidences revealed that the high-expressed CNOT7 is an oncogene with poor prognosis and participated in the progression of glioma, and HDAC2 upregulated CNOT7 expression.

In 2016, WHO applied the molecular pathological characteristics of tumors to the classification of glioma more accurately [24]. At present, molecular indicators such as IDH mutation, 1p/19q deletion, and TP53 mutation have been used for clinical detection [25]. Generally, glioma patients with chromosome 1p/19q deletion are more sensitive to radiotherapy and have a longer survival period. Patients without 1p/19q deletion usually have poor prognosis and relatively high recurrence probability [12]. Our results indicated that the CNOT7 was higher expressed in non-codel 1p/19q co-deletion status, especially in WHO grades II and III. Therefore, CNOT7 might be a potential therapeutic target for patients with non-codel 1p/19q co-deletion status in LGG.

At present, the research on the relationship between CONT7 and cancer tissues is still in the initial stage [26]. Previous studies have reported CNOT7 expression was increased in various cancers [16]. CNOT7 promoted cell proliferation, migration, and invasion of osteosarcoma cells [27]. Our studies have found that CNOT7 expression is significantly upregulated in glioma compared with normal group, including GBM and LGG. The KM curve showed that high expression of CNOT7 was associated with poor prognosis of glioma patients. It was found that CNOT7 is a candidate oncogene and poor prognostic biomarker in glioma. To date, the function of CNOT7 in glioma has not been investigated. Our work is the first attempt to explore the role of CNOT7 in glioma. The cellular experiments indicated that knockdown of CNOT7 inhibited the proliferation, migration, and invasion of glioma cell line.

CCR4-NOT protein complex is a highly conserved protein complex in eukaryotes, which has the functions of protein modification, gene transcription regulation, and depoly-adenylate tail degradation of RNA [28]. The protein complex CCR4-NOT can display different structural components, and the different subunits play different roles in biological models [29]. CNOT7 belongs to the family of DEDD adenylase, which contains RNaseD-like regions [30]. Its function of degrading mRNA and regulating gene transcription through deadenylation has been widely confirmed [13]. CNOT7 can degrade specific transcription products such as the signal products IFI27, IFI6, IFITM1, and TAP1 downstream of STAT1, and inhibit the expression of STAT1, which is a negative regulator of STAT1 [31]. Liu et al. have reported that CNOT2 negatively regulating IFN-Independent Non-Canonical JAK/STAT pathway [32]. For the first time, this study indicated the IL6-JAK-STAT3 and TNF-α signaling pathways via NF-κB were inhibited, while the CNOT7 was low-expressed. Besides, the G2M checkpoint [20] and E2F targets signaling pathways [21] were reported to promote the development of glioma. Our results revealed that the G2M checkpoint and E2F targets signaling pathways were active, while the CNOT7 was high-expressed. These evidences suggest that the CNOT7 participated in the progression of glioma via regulating glioma-related signaling pathways.

The HDAC2 (histone deacetylase 2) belongs to the histone deacetylase family, and it plays an important role in transcriptional regulation, chromatin remodeling, cell cycle progression, and development events [33]. HDAC2 removes the acetyl groups from histones, leading to a tighter packaging of chromatin and repression of gene transcription [34]. HDAC2 plays a significant role in the development of many diseases, mainly involving cancer and neurodegenerative diseases [35, 36]. HDAC2 plays an important role in the development of glioma by regulating tumor signaling pathways [37]. It has been found that HDAC2 is involved in the regulation of several important signaling pathways, including Wnt, Notch, and Hedgehog pathways [38]. In this study, we found that the HDAC2 was positive-correlated with the expression of CNOT7, HDAC2 upregulated CNOT7 expression in glioma cells. It was suspected that HDAC2 regulates CNOT7 expression by regulating downstream cancer-related signaling pathways. The discovery and development of selective HDAC2 inhibitors have great potential in the treatment of targeted cancer [39]. In addition, HDAC2 is associated with the invasion and drug resistance of glioma cells [40]. Therefore, inhibition of HDAC2 may help to reduce the invasiveness of glioma cells and improve the sensitivity to treatment.

There are some limitations in this study. First, this study is based on bioinformatics methods for analysis and interpretation. The RNA-seq data in TCGA database is the result of multi-cell level sequencing, we should carry out a single-center cohort study combined with single-cell sequencing to further optimize the current study. Second, the number of samples used in this investigation is restricted. In the future, it is necessary to further expand the sample size and carry out multicenter cohort studies, and at the same time, to conduct more in-depth mechanism and preclinical studies on the specific role of CNOT7 in glioma.

## Conclusion

In conclusion, CNOT7 is expected to be a possible therapeutic target for improving the clinical prognosis of glioma. Inhibition of CNOT or HDAC2 is a therapeutic approach for glioma treatment. This research provides reference for clinical diagnosis and treatment to explore new therapeutic targets for glioma.

## Data Availability

The datasets used or analyses during the current study are available from the corresponding author on reasonable request.
